# The Effect of chemo- and radiotherapy on tumor necrosis in soft tissue sarcoma– does it influence prognosis?

**DOI:** 10.1186/s12885-024-12027-w

**Published:** 2024-03-06

**Authors:** Julian Fromm, Alexander Klein, Maya Kirilova, Lars Hartwin Lindner, Silke Nachbichler, Boris Michael Holzapfel, Sophia Samira Goller, Thomas Knösel, Hans Roland Dürr

**Affiliations:** 1 Department of Orthopaedics and Trauma Surgery, Orthopaedic Oncology, Musculoskeletal University Center Munich (MUM), LMU University Hospital, LMU Munich, Marchioninistr. 15, D- 81377 Munich, Germany; 2 Department of Medicine III, LMU University Hospital, LMU Munich, München, Germany; 3 Department of Radiation Oncology, LMU University Hospital, LMU Munich, München, Germany; 4 Department of Radiology, LMU University Hospital, LMU Munich, München, Germany; 5 Institute of Pathology, LMU Munich, München, Germany; 6 SarKUM, Center of Bone and Soft Tissue Tumors, LMU University Hospital, LMU Munich, München, Germany

**Keywords:** Sarcoma, Surgery, Radiotherapy, Chemotherapy, Tumor necrosis, Prognosis, Predictive factor

## Abstract

**Background:**

Soft tissue sarcomas (STSs) are a heterogeneous group of tumors. Wide surgical resection is standard, often combined with neoadjuvant chemotherapy, radiotherapy, or both. Studies have shown the predictive value of tumor necrosis in bone sarcoma (BS); however, the role of necrosis in STS after neoadjuvant therapies is still unclear. This study aimed to investigate the role of chemo- and radiotherapy in the formation of tumor necrosis and to evaluate the influence of tumor necrosis on overall survival and local recurrence-free survival. Data from BS patients and patients who did not receive neoadjuvant therapy were compared.

**Methods:**

A total of 779 patients with STS or BS were treated surgically. In all patients, tumor-specific factors such as type, size, or grading and the type of adjuvant therapy were documented. Local recurrence (LR), the diagnosis of metastatic disease, and survival during follow-up were evaluated.

**Results:**

A total of 565 patients with STS and 214 with BS were investigated. In STS, 24.1% G1 lesions, 34.1% G2 lesions, and 41.8% G3 lesions were observed. Two hundred twenty-four of the patients with STS and neoadjuvant therapy had either radiotherapy (RTx) (*n* = 80), chemotherapy (CTx) (*n* = 93), or both (*n* = 51). Three hundred forty-one had no neoadjuvant therapy at all. In STS, tumor necrosis after neoadjuvant treatment was significantly higher (53.5%) than in patients without neoadjuvant therapy (15.7%) (*p* < 0.001). Patients with combined neoadjuvant chemo-/radiotherapy had substantially higher tumor necrosis than those with radiotherapy alone (*p* = 0.032). There was no difference in tumor necrosis in patients with combined chemo-/radiotherapy and chemotherapy alone (*p* = 0.4). The mean overall survival for patients with STS was 34.7 months. Tumor necrosis did not influence survival in a subgroup of G2/3 patients. In STS with no neoadjuvant therapy and grading of G2/3, the correlation between necrosis and overall survival was significant (*p* = 0.0248). There was no significant correlation between local recurrence (LR) and necrosis.

**Conclusion:**

STS shows a broad spectrum of necrosis even without neoadjuvant chemo- or radiotherapy. After CTx or/and RTx necrosis is enhanced and is significantly pronounced with a combination of both. There is a trend toward higher necrosis with CTx than with RTx. Grading substantially influences the necrosis rate, but necrosis in soft-tissue sarcoma following neoadjuvant therapy does not correlate with better survival or a lower local recurrence rate, as in bone sarcomas.

## Background

STS are a heterogeneous group of mesenchymal tumors with more than 100 histological subtypes with varying clinical behavior that make up less than 1% of all malignancies in the adult population [1]. They are found in every body part, with a predominance of the extremities, followed by the trunk [2]. The mortality of STS is as high as 40%, mainly because of distant metastasis [3]. Up to 10% of patients already show detectable metastases (most often in the lungs) at diagnosis. Wide surgical resection is commonly accepted as the therapeutic gold standard, often combined with neoadjuvant chemotherapy, radiotherapy, or both, depending on the tumor’s size, grading, and location [3]. In STS and BS, tumor necrosis is postoperatively evaluated and used as a prognostic factor. Many studies have shown the predictive value of tumor necrosis in BS, especially in osteosarcoma and Ewing sarcoma [4–6]. However, the role of posttreatment necrosis in STS is still unclear, and the data are conflicting [7]. 

Tumor necrosis is a worse prognostic factor and is part of the grading system, according to the widely used French Federation of Cancer Centers (FNCLCC) [8]. Identifying reliable prognostic criteria based on histological response is essential to enable differentiated treatment and follow-up protocols. There is no consensus concerning the prognostic value of posttreatment necrosis in STS. This study aimed to evaluate the role of chemo- and radiotherapy in forming posttreatment necrosis compared to native tumor necrosis and the influence of posttreatment necrosis on overall survival and local recurrence-free survival. We are well aware that BS and STS sarcomas are different entities with different therapy schemes and regarding BS well established neoadjuvant protocols. Necrosis has a proven impact on prognosis in BS as stated above. But, we provided the data for both to enable the reader to draw his own conclusions. Therefore, we used the data of BS patients treated in the same period and patients who did not receive neoadjuvant therapy at all.

## Methods

From 2012 to 2021, 779 consecutive patients with either STS or BS were treated surgically with curative intention at our institution. The diagnosis was preoperatively made by biopsy and postoperatively confirmed by analysis of the resection specimen. Primarily, magnetic resonance imaging (MRI) and, in some cases, computed tomography (CT) were used preoperatively to define the size and location of the tumor. A chest CT scan was obtained to assess metastatic disease. Posttreatment necrosis was quantified histologically and measured as a percentage. All patients diagnosed with STS and curative surgical therapy were included in this study. Patients without neoadjuvant therapy (either radio- or chemotherapy), surgical resection, or insufficient postoperative data were excluded.

### Surgery

Two experienced surgeons performed all the surgeries. In general, surgery was aimed at achieving an R0 resection; local or free flaps were performed as needed.

### Radiotherapy

Radiotherapy was either administered in a neoadjuvant or an adjuvant setting. The usage and timing of radiotherapy was discussed on an individual basis in multidisciplinary tumor boards.

### Chemotherapy

Chemotherapy was generally scheduled as a combined neoadjuvant and adjuvant multiagent therapy. In STS, this mainly consisted of AI (adriamycin and ifosfamide) or EIA (etoposide, ifosfamide, and adriamycin) and other regimens in some instances, including local hyperthermia [9, 10]. The usage and timing of chemotherapy was also routinely discussed in tumor boards.

### Endpoint and statistics

In all patients, tumor-specific factors such as type, size, grading, and the type of adjuvant therapy were documented. Local recurrence (LR), metastatic disease diagnosis, and patient death during follow-up were evaluated.

### Statistical analysis

All patients were followed for evidence of LR or distant metastasis in general by regional MRI scans and chest radiographs or CT scans. Clinical outcomes of LR, LR-free survival (LRFS), and overall survival (OS) were used for assessment. LRFS and OS were defined as the time from surgery to the first occurrence of local recurrence or death from any cause. For statistical analysis, overall and local recurrence-free survival were calculated using the Kaplan‒Meier method. Significance analysis was performed using the log-rank test, the chi-square test, or the Cox proportional hazards regression model. A p-value of less than 0.05 was considered statistically significant. The data analysis software used was MedCalc® (MedCalc Software, Ostend, Belgium) and SPSS 24®.

## Results

### Patient characteristics

Of the 411 men and 368 women (total *n* = 779), 565 had STS and 214 had bone sarcoma (BS). The mean age at surgery was 55 years (2.6–99.7 years), with a mean age of 41.4 years for patients with BS and 60.3 years for patients with STS (*p* < 0.001). The mean follow-up was 35 months (0–106 months). The most common diagnosis in STS was liposarcoma in 29.1% (*n* = 164) of the patients, followed by undifferentiated pleomorphic sarcoma (UPS) in 27.5% (*n* = 155) (Table 1).

**Table 1:** Distribution of histotypes

Tumor Entity	N	%
**Soft tissue sarcoma**	**565**	
Liposarcoma	164	29.1
Undifferentiated pleomorphic sarcoma (UPS)	155	27.5
Myxofibrosarcoma	60	10.6
Leiomyosarcoma	42	7.4
Synovial sarcoma	34	6.0
Malignant Peripheral Nerve Sheath Tumor	25	4.4
Fibrosarcoma	14	2.5
Clear cell sarcoma	12	2.1
Other	59	10.5
**Bone sarcoma**	**214**	
Chondrosarcoma	83	38.8
Osteosarcoma	82	38.3
Ewing sarcoma	30	14.0
Other	19	8.9

The most common diagnosis in patients with BS was chondrosarcoma in 38.8% (*n* = 83) and osteosarcoma in 38.3% (*n* = 82) of cases. In most cases, the tumor was located in the thigh, followed by the lower leg in both STS and BS.

A total of 86.0% of STSs (*n* = 486) were located deep in the tissue compared to 99.5% of BSs (*n* = 213) (*p* < 0,001). In 10 patients with STS, there was no grading possible. Of the remaining 555 patients, 24.1% had grade 1 (*n* = 134), 34.1% had grade 2 (*n* = 189), and 41.8% had grade 3 (*n* = 232) lesions. Of the 214 patients with BS, 15.9% had a grade 1 (*n* = 34), 29.4% a grade 2 (*n* = 63), and 54.7% a grade 3 (*n* = 117) lesion. There was no statistical correlation between superficial growth or deeper growth and grading of the lesion in either STS (*p* = 0.077) or BS (*p* = 0.3). The mean tumor size was 8.9 cm in patients with STS and 8.2 cm in patients with BS (*p* = 0.172). In patients with STS, R0 resection was achieved in 454 lesions (80.0%), R1 resection in 106 (19.0%), and R2 resection in 5 lesions (1.0%). An R1 resection was intended in most patients, especially atypical lipomas (liposarcoma G1 of the extremities) in 61 cases. Those lesions seem to have a more benign behaviour and are therefore treated differently [11]. There were 194 (91.0%) R0 resections in BS and 20 (9.0%) R1 resections. There were no R2 resections.

### Tumor necrosis

Four hundred sixty-five patients did not have any form of neoadjuvant therapy. A total of 224 (40.0%) of the patients with STS had either neoadjuvant radiotherapy (*n* = 80, 14.0%), chemotherapy (*n* = 93, 17.0%), or both (*n* = 51, 9.0%). Ninety patients (42.0%) with BS had either neoadjuvant radiotherapy (*n* = 1, 0.5%), chemotherapy (*n* = 81, 40.0%), or both (*n* = 8, 4.0%). In neoadjuvantly treated patients, the mean tumor necrosis was 53.5% (range 0–100%) in STS and 57.7% (range 0–100%) in BS (*p* = 0.33). Consequently, both groups had a mean Salzer-Kuntschik [12] grade of 4. Details are shown in Table 2.

**Table 2:** Tumor necrosis concerning therapy

	N	Necrosis (%)	G1	G2	G3
**Soft tissue sarcoma**			N (Necrosis %)		
No neoadjuvant therapy	341	15.7	110 (4.1)	114 (14.8)	109 (28.4)
Chemotherapy	93	52.7	3(32.0)	30 (39.6)	60 (60.3)
Radiotherapy	80	45.7	19 (39.5)	27 (39.3)	34 (54.3)
Chemo- and radiotherapy	51	67.1	2 (75.0)	18 (62.4)	29 (70.6)
			**134 (10.8)**	**189 (26.7)**	**117 (49.7)**
**Bone sarcoma**					
No neoadjuvant therapy	124	20.9	34 (6.5)	49 (23.0)	41 (30.4)
Chemotherapy	81	56.7		11 (40.6)	70 (59.2)
Radiotherapy	1	100		1 (100)	
Chemo- and radiotherapy	8	62.8		2 (37.5)	6 (71.2)
			**34 (6.5)**	**63 (27.7)**	**117 (49.7)**

In STS, tumor necrosis after therapy was significantly higher than in patients without neoadjuvant therapy (*p* < 0.001). Patients with combined neoadjuvant chemo-/radiotherapy had significantly higher tumor necrosis than those with radiotherapy alone (*p* = 0.032). Nevertheless, there was no difference in tumor necrosis in patients with combined radio-/chemotherapy and chemotherapy alone (*p* = 0.4). Size was also a significant factor. Of all STS patients those with tumors ≤ 5 cm (*n* = 229) had a mean necrosis of 26.7% compared to 33.4% in larger ones (*n* = 336; 0.0204). Comparing deep and superficial tumors, were was a significant difference of 33.2% vs. 15.2% (*p* = 0.004). In a multiple regression analysis grading, size and location kept significant. Including neoadjuvant therapy of any form (CTX or RTX), size lost its significance.

Tumor necrosis was significantly lower in patients with BS without neoadjuvant therapy than in those who received neoadjuvant therapy (*p* < 0.001). In STS and BS, tumor necrosis was increased with higher tumor grading (*p* < 0.001).

### Survival and local recurrence

A total of 10.1% (*n* = 57) of patients with STS had metastatic disease (MD) at the time of diagnosis, compared to 10.3% (*n* = 22) of patients with BS (n. s.). A total of 23.7% (*n* = 134) of patients with STS developed MD during observation, as did 24.8% (*n* = 53) of patients with BS. In STS, 84 patients with a grade 3 lesion developed MD, compared to 45 patients with grade 2 lesions and 2 patients with grade 1 lesions (*p* < 0.001). In BS, 42 patients with grade 3 lesions developed MD, compared to 10 patients with grade 2 lesions and 1 patient with a grade 1 lesion (*p* < 0.001). A total of 11.5% (*n* = 65) of the patients with STS developed local recurrence, as did 16.4% (*n* = 35) of the patients with BS (n.s.). A total of 24.4% (*n* = 138) of patients with STS died during the observation period, as did 18.7% (*n* = 40) of patients with BS (n.s. ).

The mean overall survival for patients with STS was 34.7 months (range 0–106 months) compared to 37.2 months (0.5–106 months) in patients with BS (not significant). In STS and BS, grading was a highly significant predictor for worse survival (*p* < 0.001), as well as age (*p* < 0.001) and metastatic disease (*p* < 0.001) during the follow-up. In BS and STS, a tumor size over 8 cm was associated with worse survival (*p* = 0.001 and *p* = 0.002).

In BS, tumor necrosis below 90.0% was associated with significantly worse survival (*p* = 0.04); however, in STS, there was no difference (Table 3). Fifty-five of 431 (12.8%) patients with STS and tumor necrosis below 90%, as well as 15 out of 134 (11.2%) patients with tumor necrosis above 90%, developed MD (n.s. ).

**Table 3:** Multivariate analysis showing no statistical significance in the correlation of overall survival and tumor necrosis in soft tissue sarcoma. HR indicates hazards ratio; CI 95% confidence interval

	P	HR	95% CI of Exp (b)
Grading	**< 0.0001**	2.4541	1.8171 to 3.3144
Metastasis	**< 0.0001**	4.4960	2.9952 to 6.7488
Age	**< 0.0001**	1.0279	1.0155 to 1.0405
Deep location	0.3144	1.3689	0.7424 to 2.5242
Tumor size	**0.0032**	1.0327	1.0108 to 1.0550
Necrosis	0.9491	0.9998	0.9945 to 1.0052

In BS, neoadjuvant therapy was associated with better overall survival (*p* = 0.008); however, in STS, there was no statistical significance (*p* > 0.5). In our population, patients with BS and chemotherapy had a significantly better OS than patients without chemotherapy (*p* < 0.039). There was no statistical correlation between OS and the type of neoadjuvant therapy in STS.

**Fig. 1:**
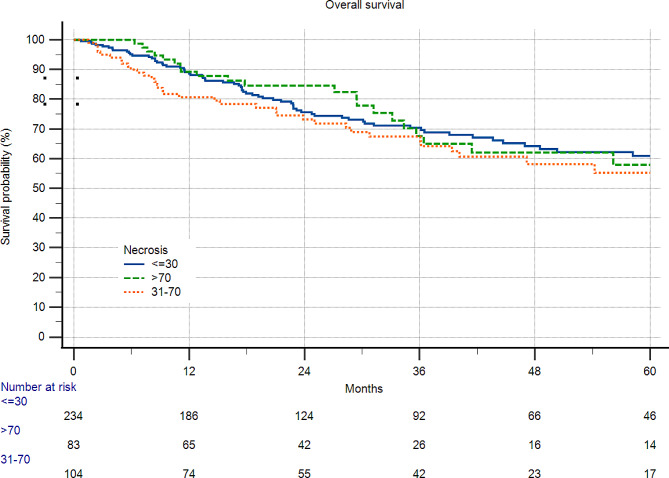
421 patients with soft tissue sarcoma and grade 2/3 disease. Patients without neoadjuvant therapy were included. There was no difference between the rate of necrosis and survival

**Fig. 2:**
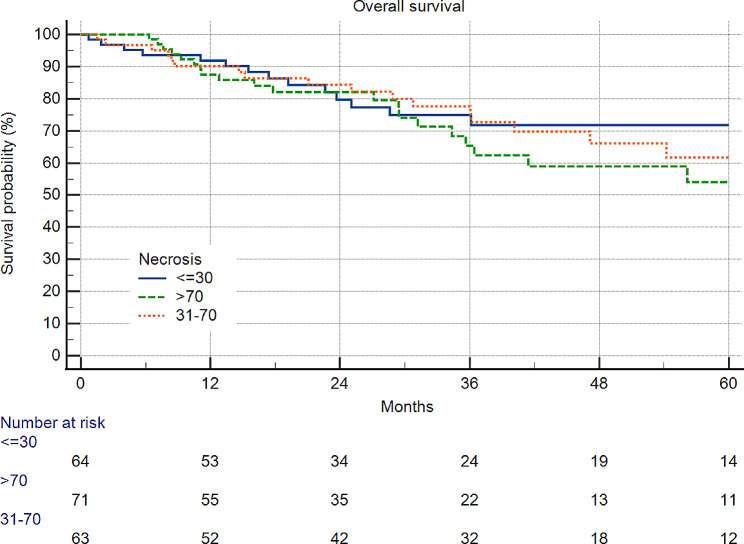
198 patients with soft tissue sarcoma and grading 2/3. Patients without neoadjuvant therapy were excluded. There was also no difference between the rate of necrosis and survival

In STS, a subgroup of G2/3 patients categorized with tumor necrosis did not influence survival (Figs. 1 and 2).

**Fig. 3:**
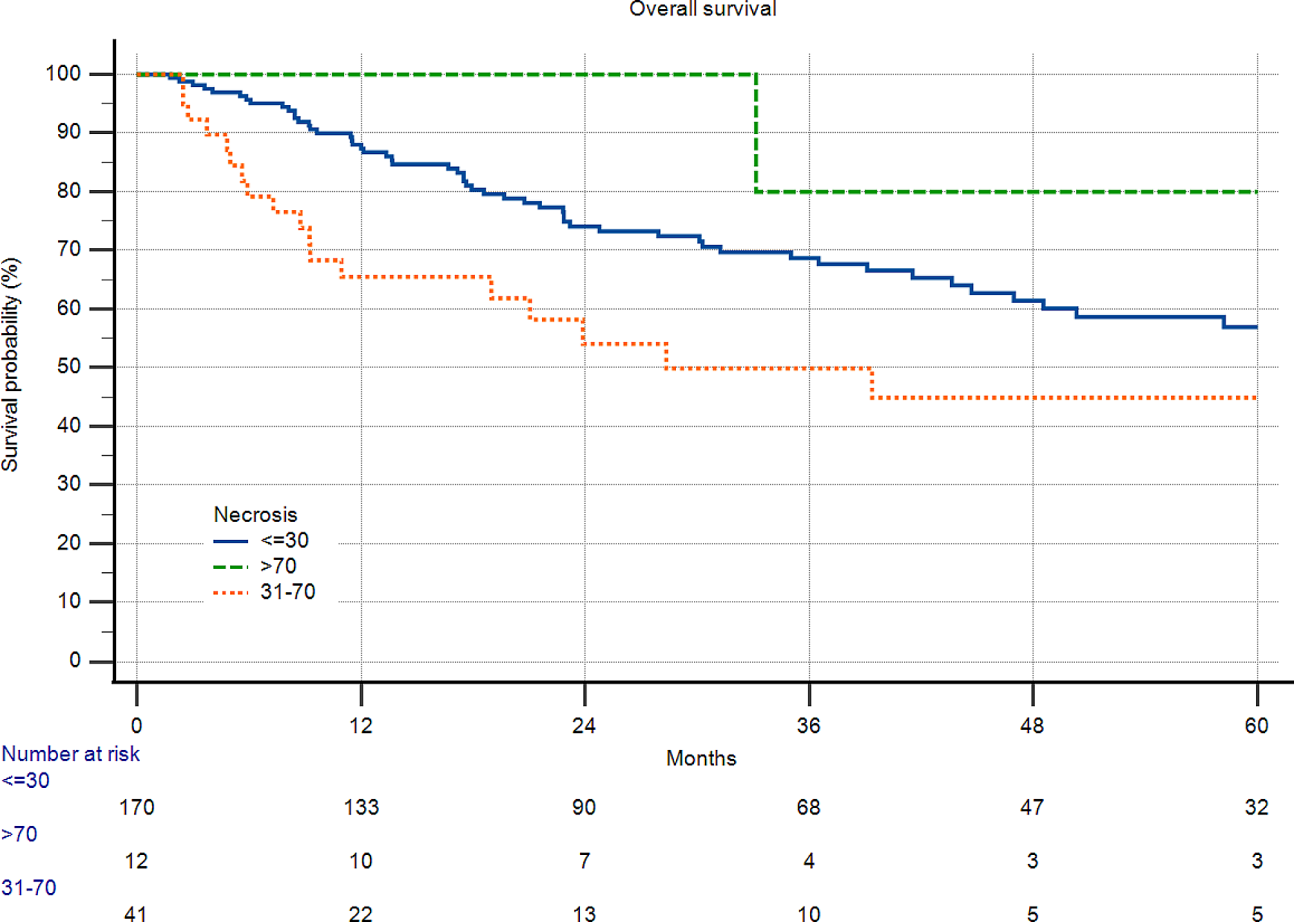
A total of 223 patients with soft tissue sarcoma and grade 2/3 disease. Only patients without neoadjuvant therapy. There was a significant correlation between necrosis and survival (*p* = 0.0248)

As a control, we investigated in STS the correlation of necrosis and overall survival in those patients with no neoadjuvant therapy and a grading of G2/3, which was, as expected, significant (*p* = 0.0248) (Fig. 3).

In STS, as in BS, there was no significant correlation between LR and age, location, grading, or resection status. However, there was a significant correlation between LR and R1-resected lesions (11 of 20; 55.0%) when compared to R0-resected lesions (26 of 168; 16.5%) (*p* < 0.001).

Local recurrence was more common in patients with BS and tumor necrosis below 90% (*n* = 34) than in patients with tumor necrosis over 90.0% (*n* = 1) (*p* = 0.029), but there was no statistical significance in patients with STS. Local recurrence during the follow-up period was correlated with worse survival in patients with STS (*p* < 0.005) as well as in patients with BS (*p* = 0.001). There was no correlation between tumor size and LR.

## Discussion

STS are a common and diverse group of tumors with different histological subtypes. Treatment consists of surgical resection and, in many cases, (neo)adjuvant chemo- or radiotherapy. In this study, the most common diagnosis was liposarcoma, followed by UPS, which is in accordance with the literature [1]. Age, grading, metastatic disease, and a tumor size greater than 8 cm were highly significant predictors for worse survival in STS [13]. Native tumor necrosis is also a strong independent predictor of an unfavorable disease either seen on CT, MRI, or, more recently, on positron emission tomography (PET) scans or in histopathology [14–16]. Even if the threshold is as low as 10% necrosis, this worsens the prognosis [17]. Therefore, tumor necrosis has been included in the grading system since the 1980s [8]. 

Neoadjuvant therapy is part of advanced or high-grade sarcomas. Neoadjuvant chemotherapy may prolong disease-free survival but is still in debate for overall survival benefits [18]. Our data support a survival benefit in high-risk groups. Hence, adjuvant chemotherapy is part of our treatment protocol [9]. 

Necrosis measured at the resected specimen is used as a marker for the effectiveness of neoadjuvant therapy in BS [19]. This contrasts with STS, and the data are conflicting.

As expected, our patients had significantly higher tumor necrosis after receiving neoadjuvant therapies. Overall, there was no difference between tumor necrosis after receiving radio- or chemotherapy. However, there was significantly higher necrosis after receiving both radio- and chemotherapy compared to radiotherapy alone. A higher grading was connected to higher tumor necrosis, which could be because there is a higher cellular turnover or because, by definition, the grading system used higher spontaneous necrosis in high-grade tumors [8]. 

Shah et al. published 30 patients with STS and neoadjuvant RTx with a mean tumor necrosis of 35%, which aligns with our findings. Due to a 100% distant recurrence-free survival in three patients with 100% tumor necrosis after RTx compared to 63% in the remaining 27 patients, they postulated that at least a complete pathologic response is predictive of oncological outcome [20]. Tumor necrosis after RTx is somewhat dependent on grading, as shown in Table 2. In addition, other authors, such as Kaiser et al., described that in an even more noticeable finding with 67.7% in G2/3 and 14.7% in G1 tumors [21]. This has to be considered in assessing investigations with inhomogeneous patient cohorts, as often seen in the literature.

In 2006, Menendez et al. published a series of 82 patients who had neoadjuvant CTx with an average necrosis of 64%. Thirty-two patients had tumor necrosis ≥ 95%. These patients showed no survival benefit compared to patients with less necrosis [22]. They concluded that tumor necrosis after CTx does not predict the outcome, as seen also in our data. In contrast, Gannon et al. published 162 patients with STS, 123 of whom had high-grade STS. All had neoadjuvant RTx, and 56 of them also had neoadjuvant CTx. They found an average necrosis of 25.0%. In summary, patients with higher than 10% necrosis had worse overall and progression-free survival. In tumors equal to or greater than 10 cm, necrosis lost significance. This underlines necrosis as a prognostic factor, but it was not investigated concerning neoadjuvant therapy [7]. 

One of the most important studies published in 2001 in this context is Eilber et al. from UCLA in California [23]. A total of 496 patients with G2/3 STS received various combinations of doxorubicin, cisplatin, and ifosfamide in a neoadjuvant setting. In addition, they received neoadjuvant RTx. The authors decided to classify necrosis into two groups with a threshold of 95%. 38% of the patients had no residual tumor, 14% had ≥ 95%, and 48% had < 95% necrosis. Overall survival was 71% at five years, comparable with this study; patients with ≥ 95% necrosis had a more extended five-year-OS (80%) than those with < 95% (62%). No residual tumor proved to have the same outcome as ≥ 95% necrosis. Mullen et al. investigated CTx and RTx-induced necrosis in 113 G2/3 STS patients at the MGH in Boston [24]. All patients received both modalities. 44% of the patients had ≥ 95% necrosis; the median necrosis was 90%. LR, 5-year disease-specific survival, and OS were not different between the groups. We compared this to our data. In G2/3 patients with both CTx and RTx (*n* = 47), 25 (53%) had ≥ 90% necrosis, and OS was > 90% and not different in either group. The authors concluded that the extent of therapy-induced tumor necrosis did not correlate with outcome.

In contrast, in a tiny group (*n* = 34) of patients with advanced STS and combined neoadjuvant CTx and RTx, the authors observed 50% of the cohort with tumor necrosis ≥ 90% [25]. A higher rate of necrosis (> 90%) showed a trend toward better five-year survival (67.3% vs. 26.9%). However, one must keep in mind the inhomogeneous cohort of those patients.

This is in line with recently published studies by Weiss et al. including 81 patients with large (≥ 5 cm) and high-risk (G2/3) STS. Neoadjuvant CTX with ifosfamide and doxorubicin in addition to 45 Gy of radiation resulted in a rate of necrosis of more than 90% in 22% of the patients. By adding pazopanib in a randomized fashion this could be significantly raised to 58% [26]. Published 3 years later, 3-year event-free survival and overall survival was not significantly different. But there was a trend towards better OS in the group with higher necrosis [27]. The authors concluded that pathologic necrosis may not accurately measure treatment response but may reflect the tumor biology itself.

In patients with BS in our cohort, there was a significant correlation between tumor necrosis ≥ 90% and better overall survival, which is consistent with the literature. While no evidence supports this in chondrosarcomas, the correlation between tumor necrosis and overall survival has been established in osteosarcoma and Ewing sarcoma for decades.

However, there was no correlation between tumor necrosis and OS, LR, or MD in patients with STS. These data and the literature imply that, as opposed to patients with BS, in STS, we cannot look for tumor necrosis as a marker for the success of neoadjuvant therapy. Some results, as described above in patients with near-total or total tumor necrosis after combined neoadjuvant CTx and RTx, had to be seen as exceptional findings.

Measuring the effect of RTx and CTx in a single parameter, such as tumor necrosis, might be too short-sighted for malignancies as complex as sarcomas, with several subtypes and sometimes unpredictable biology. However, in another study conducted in our hospital using some of the patients in this study, PET-CT scans performed before and after neoadjuvant CTx focusing specifically on functional parameters, not on necrosis, showed a better progression-free survival in those patients with a reduction in metabolism after CTx [28]. 

Regarding local recurrence, the correlation between tumor necrosis after neoadjuvant therapy and LR is unclear, and the literature is conflicting. In this study, there was no significant connection in patients with STS. Interestingly, the resection margin did not show a significant correlation with LR. The relationship between the R-status and LR, the best margin to be obtained, and the need for a wide resection after accidental, incomplete R1 resections is highly disputed [29]. 

### Limitations of the study

This study has a retrospective design. The patients had been registered prospectively, and pathologic investigation was also performed prospectively. Only sarcoma patients were included, but those with all subspecialties were included. For better comparison, we used subgroups regarding grading and different neoadjuvant forms of therapy.

Focusing on therapy-induced necrosis, we used patients without neoadjuvant therapy as a comparison. However, this group is different from the therapy group, with less aggressive tumors in older patients, so the results have to be compared with that in mind.

Furthermore, using BS as a second comparison group opens a wide door of inhomogeneity in this entity. Nevertheless, even with that, we could prove some principal differences in the induction of tumor necrosis compared to STS.

## Conclusions

STS shows a wide spectrum of necrosis even without neoadjuvant chemo- or radiotherapy. After CTx and/or RTx, necrosis is enhanced and significantly pronounced with a combination of both. There is a trend toward higher necrosis with CTx than with RTx. Grading substantially influences the rate of necrosis. However, a higher rate of necrosis does not correlate with a better outcome with respect to survival or local recurrence, as in bone sarcomas.

## Data Availability

The datasets used and analyzed during the current study are available from the corresponding author upon reasonable request.

## Abbreviations

BS: bone sarcoma
CI 95%: confidence interval
CT: computed tomography
CTx: chemotherapy
G1, G2, G3: grading according to the French Federation of Cancer Centers grading system
HR: hazards ratio
LR: local recurrence
LRFS: local recurrence-free survival
MD: metastatic disease
MRI: magnetic resonance imaging
OS: overall survival
PET: positron emission tomography
R0, R1, R2: resection margin
RTx: radiotherapy
STS: soft tissue sarcoma

## References

[1] Board E (2020). Soft tissue and bone tumours.

[2] Arifi S, Belbaraka R, Rahhali R, Ismaili N (2015). Treatment of adult soft tissue sarcomas: an overview. Rare Cancers Ther.

[3] Gronchi A, Miah AB, Dei Tos AP, Abecassis N, Bajpai J, Bauer S, Biagini R, Bielack S, Blay JY, Bolle S (2021). Soft tissue and visceral sarcomas: ESMO-EURACAN-GENTURIS clinical practice guidelines for diagnosis, treatment and follow-up(☆). Ann Oncol.

[4] Bielack SS, Kempf-Bielack B, Delling G, Exner GU, Flege S, Helmke K, Kotz R, Salzer-Kuntschik M, Werner M, Winkelmann W (2002). Prognostic factors in high-grade osteosarcoma of the extremities or trunk: an analysis of 1,702 patients treated on neoadjuvant cooperative osteosarcoma study group protocols. J Clin Oncol.

[5] Bacci G, Ferrari S, Bertoni F, Rimondini S, Longhi A, Bacchini P, Forni C, Manfrini M, Donati D, Picci P (2000). Prognostic factors in nonmetastatic Ewing’s sarcoma of bone treated with adjuvant chemotherapy: analysis of 359 patients at the Istituto Ortopedico Rizzoli. J Clin Oncol.

[6] Sindhu II, Mehreen A, Wali RM, Abubakar M (2021). Clinical outcome of paediatric ewing sarcoma and significance of pathological necrosis for mortality after neoadjuvant chemotherapy: single institutional study. J Pak Med Assoc.

[7] Gannon NP, Stemm MH, King DM, Bedi M (2019). Pathologic necrosis following neoadjuvant radiotherapy or chemoradiotherapy is prognostic of poor survival in soft tissue sarcoma. J Cancer Res Clin Oncol.

[8] Trojani M, Contesso G, Coindre JM, Rouesse J, Bui NB, de Mascarel A, Goussot JF, David M, Bonichon F, Lagarde C (1984). Soft-tissue sarcomas of adults; study of pathological prognostic variables and definition of a histopathological grading system. Int J Cancer.

[9] Issels RD, Lindner LH, Verweij J, Wessalowski R, Reichardt P, Wust P, Ghadjar P, Hohenberger P, Angele M, Salat C (2018). Effect of Neoadjuvant Chemotherapy Plus Regional Hyperthermia on Long-Term outcomes among patients with localized high-risk soft tissue sarcoma: the EORTC 62961-ESHO 95 Randomized Clinical Trial. JAMA Oncol.

[10] Issels RD, Lindner LH, Verweij J, Wust P, Reichardt P, Schem BC, Abdel-Rahman S, Daugaard S, Salat C, Wendtner CM (2010). Neo-adjuvant chemotherapy alone or with regional hyperthermia for localised high-risk soft-tissue sarcoma: a randomised phase 3 multicentre study. Lancet Oncol.

[11] Rauh J, Klein A, Baur-Melnyk A, Knosel T, Lindner L, Roeder F, Jansson V, Dürr HR (2018). The role of surgical margins in atypical Lipomatous tumours of the extremities. BMC Musculoskelet Disord.

[12] Salzer-Kuntschik M, Brand G, Delling G (1983). [Determination of the degree of morphological regression following chemotherapy in malignant bone tumors]. Pathologe.

[13] Callegaro D, Miceli R, Bonvalot S, Ferguson P, Strauss DC, Levy A, Griffin A, Hayes AJ, Stacchiotti S, Pechoux CL (2016). Development and external validation of two nomograms to predict overall survival and occurrence of distant metastases in adults after surgical resection of localised soft-tissue sarcomas of the extremities: a retrospective analysis. Lancet Oncol.

[14] Gustafson P, Herrlin K, Biling L, Willen H, Rydholm A (1992). Necrosis observed on CT enhancement is of prognostic value in soft tissue sarcoma. Acta Radiol.

[15] Rakheja R, Makis W, Tulbah R, Skamene S, Holcroft C, Nahal A, Turcotte R, Hickeson M (2013). Necrosis on FDG PET/CT correlates with prognosis and mortality in sarcomas. AJR Am J Roentgenol.

[16] Lack EE, Steinberg SM, White DE, Kinsella T, Glatstein E, Chang AE, Rosenberg SA (1989). Extremity soft tissue sarcomas: analysis of prognostic variables in 300 cases and evaluation of tumor necrosis as a factor in stratifying higher-grade sarcomas. J Surg Oncol.

[17] Lin CN, Chou SC, Li CF, Tsai KB, Chen WC, Hsiung CY, Yen CF, Huang HY (2006). Prognostic factors of myxofibrosarcomas: implications of margin status, tumor necrosis, and mitotic rate on survival. J Surg Oncol.

[18] Smolle MA, Andreou D, Tunn PU, Szkandera J, Liegl-Atzwanger B, Leithner A (2017). Diagnosis and treatment of soft-tissue sarcomas of the extremities and trunk. EFORT Open Rev.

[19] Bacci G, Longhi A, Versari M, Mercuri M, Briccoli A, Picci P (2006). Prognostic factors for osteosarcoma of the extremity treated with neoadjuvant chemotherapy: 15-year experience in 789 patients treated at a single institution. Cancer.

[20] Shah D, Borys D, Martinez SR, Li CS, Tamurian RM, Bold RJ, Monjazeb A, Canter RJ (2012). Complete pathologic response to neoadjuvant radiotherapy is predictive of oncological outcome in patients with soft tissue sarcoma. Anticancer Res.

[21] Kaiser D, Schelm M, Gerber C, Brown ML, Muller DA (2021). The effect of preoperative radiotherapy on surgical resectability, tumor volume and the necrosis rate of soft tissue sarcomas: a retrospective single-center analysis. Surg Oncol.

[22] Menendez LR, Ahlmann ER, Savage K, Cluck M, Fedenko AN (2007). Tumor necrosis has no prognostic value in neoadjuvant chemotherapy for soft tissue sarcoma. Clin Orthop Relat Res.

[23] Eilber FC, Rosen G, Eckardt J, Forscher C, Nelson SD, Selch M, Dorey F, Eilber FR (2001). Treatment-induced pathologic necrosis: a predictor of local recurrence and survival in patients receiving neoadjuvant therapy for high-grade extremity soft tissue sarcomas. J Clin Oncol.

[24] Mullen JT, Hornicek FJ, Harmon DC, Raskin KA, Chen YL, Szymonifka J, Yeap BY, Choy E, DeLaney TF, Nielsen GP (2014). Prognostic significance of treatment-induced pathologic necrosis in extremity and truncal soft tissue sarcoma after neoadjuvant chemoradiotherapy. Cancer.

[25] MacDermed DM, Miller LL, Peabody TD, Simon MA, Luu HH, Haydon RC, Montag AG, Undevia SD, Connell PP (2010). Primary tumor necrosis predicts distant control in locally advanced soft-tissue sarcomas after preoperative concurrent chemoradiotherapy. Int J Radiat Oncol Biol Phys.

[26] Weiss AR, Chen YL, Scharschmidt TJ, Chi YY, Tian J, Black JO, Davis JL, Fanburg-Smith JC, Zambrano E, Anderson J (2020). Pathological response in children and adults with large unresected intermediate-grade or high-grade soft tissue sarcoma receiving preoperative chemoradiotherapy with or without pazopanib (ARST1321): a multicentre, randomised, open-label, phase 2 trial. Lancet Oncol.

[27] Weiss AR, Chen YL, Scharschmidt TJ, Xue W, Gao Z, Black JO, Choy E, Davis JL, Fanburg-Smith JC, Kao SC (2023). Outcomes after preoperative chemoradiation with or without Pazopanib in Non-rhabdomyosarcoma Soft tissue sarcoma: a Report from Children’s Oncology Group and NRG Oncology. J Clin Oncol.

[28] Fendler WP, Lehmann M, Todica A, Herrmann K, Knösel T, Angele MK, Dürr HR, Rauch J, Bartenstein P, Cyran CC (2015). PET response criteria in solid tumors predicts progression-free survival and time to local or distant progression after chemotherapy with regional hyperthermia for soft-tissue sarcoma. J Nucl Med.

[29] Bilgeri A, Klein A, Lindner LH, Nachbichler S, Knösel T, Birkenmaier C, Jansson V, Baur-Melnyk A, Dürr HR. The Effect of Resection Margin on Local Recurrence and Survival in High Grade Soft Tissue Sarcoma of the Extremities: How Far Is Far Enough? Cancers (Basel) 2020, 12(9). 10.3390/cancers12092560 PMC7563962 32911853

